# Sensing Technologies for Detection of Acetone in Human Breath for Diabetes Diagnosis and Monitoring

**DOI:** 10.3390/diagnostics8010012

**Published:** 2018-01-31

**Authors:** Valentine Saasa, Thomas Malwela, Mervyn Beukes, Matlou Mokgotho, Chaun-Pu Liu, Bonex Mwakikunga

**Affiliations:** 1 DST/CSIR, PO BOX 395, Pretoria 0001, South Africa; tmalwela@csir.co.za; 2 Departmentof Biochemistry, University of Pretoria, Pretoria 0001, South Africa; mervyn.beukes@up.ac.za; 3 Department of Biochemistry, University of Limpopo, P/Bag x1106, Sovenga 0727, South Africa; Matlou.mokgotho@ul.ac.za; 4 Department of Materials Science and Engineering, National Cheng Kung University, Tainan 70101, Taiwan; cpliu@mail.ncku.edu.tw

**Keywords:** diabetes mellitus, breath analysis, non-invasive diagnosis, nanomaterials, chemoresistivesensors, acetone detection

## Abstract

The review describes the technologies used in the field of breath analysis to diagnose and monitor diabetes mellitus. Currently the diagnosis and monitoring of blood glucose and ketone bodies that are used in clinical studies involve the use of blood tests. This method entails pricking fingers for a drop of blood and placing a drop on a sensitive area of a strip which is pre-inserted into an electronic reading instrument. Furthermore, it is painful, invasive and expensive, and can be unsafe if proper handling is not undertaken. Human breath analysis offers a non-invasive and rapid method for detecting various volatile organic compounds thatare indicators for different diseases. In patients with diabetes mellitus, the body produces excess amounts of ketones such as acetoacetate, beta-hydroxybutyrate and acetone. Acetone is exhaled during respiration. The production of acetone is a result of the body metabolising fats instead of glucose to produce energy. There are various techniques that are used to analyse exhaled breath including Gas Chromatography Mass Spectrometry (GC–MS), Proton Transfer Reaction Mass Spectrometry (PTR–MS), Selected Ion Flow Tube-Mass Spectrometry (SIFT–MS), laser photoacoustic spectrometry and so on. All these techniques are not portable, therefore this review places emphasis on how nanotechnology, through semiconductor sensing nanomaterials, has the potential to help individuals living with diabetes mellitus monitor their disease with cheap and portable devices.

## 1. Introduction

The detection of acetone in diabetes-affected breath strengthens the possibility for successful treatment and also maintains the demand for cheap, non-invasive and quantitative diagnosis of diabetes mellitus. People living with type 1 and type 2 diabetes mellitus are required to monitor their disease daily; at least twice a day in order to manage and monitor the blood glucose level. Diabetes mellitus (DM) is a chronic metabolic disorder resulting from insulin deficiency [1]. It may be due to insufficient insulin secretion or defects in insulin action, which result in a syndrome of mainly sugar, protein and lipid metabolic disorders [2]. Insulin is a hormone that converts sugar, starches and other food into energy [3]. All forms of diabetes are characterized by hyperglycemia, a defect or absence of insulin, the development of diabetes-specific pathology in the retina, renal glomerulus and peripheral nerve, microvascular (such as retina, renal glomerulus and peripheral nerve) and macrovascular (such as atherosclerosis), coronary artery disease and stroke [4,5]. The signs and symptoms of hyperglycemia include polyuria, polydipsia, weight loss, blurred vision and hypotension [1]. There are two common forms of diabetes mellitus; type1 diabetes, which is characterized by an early stage of onset and inability to produce insulin, andtype 2 diabetes, which is characterized by a late stage of onset and inability to utilize insulin properly [6].

Diabetes mellitus is a manageable disorder, in which the level of blood glucose is monitored daily. The diagnosis and monitoring of blood glucose as well as ketone bodies currently used in clinical studies on diabetic and prediabetes patients involve the use of blood tests [7]. This is done by drawing blood from a patient and using a typical Accuchek™ (Roche, Indianapolis, Indiana glucometer. This glucometer measurement involves pricking for a drop of blood and placing such a drop on a sensitive area of a strip which is pre-inserted in an electronic reading instrument. This method is painful, invasive and can be unsafe. Therefore, it does not suit everyone, especially in the case where the patient needs several samplings each day.

The non-invasive technologies, more especially the nanosensorbreath technologies which are portable, cheap to fabricate, highly sensitive and easy to use, have potential in diabetes monitoring. According to the International Diabetes Federation (IDF), diabetes will be the seventhleading cause of death in 2030 and the number of diabetes patients will remarkably increase by more than 50% worldwide by 2030 when compared to the number in 2011. Therefore, there is a high demand for a better and pain-free method of monitoring both type 1 and type 2 diabetes mellitus. Managing and monitoring the blood glucose reduces the risk of diabetic coma and ultimately the death rate. Breath acetone has been shown to have strong correlation with blood glucose level [8,9,10,11,12,13,14,15,16]. According to the World Bank, economic growth in Africa has been expanding robustly over the last decade. In 2012, the growth of sub-Saharan Africa (SSA) was estimated to be 4.7% overall, and 5.8% excluding South Africa, the largest economy of the region.A growth in the economy directly impacts the lifestyle of the people, and this is evident in the diet-related issues such as diabetes mellitus [17].Thus, there is a great potential need of using breath acetone as a biomarker for diabetes mellitus, and detect it using non-invasive chemoresistive sensors.

Human exhaled breath contains thousands of different volatile organic compounds (VOCs) derived from the body’s metabolic processes. In patients with diabetes mellitus, the body produces excess amounts of ketones such as acetone because the body uses fats instead of glucose to produce energy, which are then exhaled during respiration [18]. Acetone has been successfully used as a biomarker for diabetes mellitus, especially in type 1 diabetes mellitus [19]. During fasting, exercise and or diabetes mellitus, the liver produces ketone bodies to act as an additional energy source, which are later metabolized into acetone and other ketone bodies. The acetone that is produced travels through the blood and is excreted through urine or exhaled breath. For the exhaled breath, it has been found that the partition coefficient is 330 parts in the blood for every one part that leaves with expired air [20]. It has been found that quantification of acetone concentration in human breath, using breath analysis techniques, correlates strongly with acetone concentration in the blood and other ketone bodies such as beta-hydroxybutyrate. Thus, measurement of acetone from breath gives better diagnostic control of a patient’s diabetic condition, rather than through the use of blood glucose measurements alone.

Many studies have reported on non-invasive analysis of breath for detection of diseases using techniques such as gas chromatography with mass spectrometry (GC–MS), proton transfer reaction with mass spectrometry (PTR–MS) and many other sensitive techniques [3,21,22,23,24]. Although these techniques are sensitive and reliable, they are not portable for daily monitoring. Therefore we are going to outline the advantages of the nano-based sensors for detection of VOCs, more especially for acetone over other techniques that are used.

## 2. Human Breath for Diagnosis of Diseases

Human breath is mainly composed of oxygen, nitrogen, carbon dioxide, water and so on. The volatile organic compounds, described fully in the section below, are derived in the body or from the environment, and make up the rest of the breath [25]. Human breath has been used as a potential tool for the diagnosis and study of diseases. The human breath was first analyzed by Pauling in 1971 [26] using gas chromatography and around 250 different gases were identified. Currently, scientists are able to detect more than 300 different volatile organic compounds and other particles in breath [27]. Thus, human breath analysis is also crucial for delivering non-invasive, real-time and rapid screening and diagnosis of complex diseases such as cancers and acute infections [28]. Furthermore, it is not only non-invasive, but also has several advantages as compared to traditional diagnostic techniques, which include painless procedures and sampling that does not require skilled medical staff [29].

The human breath contains several hundred VOCs with concentrations ranging from part-per-trillion (ppt) to part-per-million (ppm). The cellular and biochemical origin of many of these VOCs has not been determined and some of them might be of exogenous origin. The acetone concentration in the breath varies from 300 to 900 ppb in healthy people to more than 1800 ppb in individuals with diabetes [18]. Therefore, acetone can act as a biomarker for metabolic (diabetes) conditions in the bloodstream. In certain cases such as fasting, exercising and being diabetic, the liver produces ketones to act as an additional energy source, which are then metabolized into acetone and other ketone bodies. Using breath analysis techniques, acetone concentrations in exhaled breath have been shown to correlate with the acetone concentrations in the blood as well as with other ketones such as beta-hydroxybutyrate. In addition, it is also found that the level of blood glucose can be correlated to the volatile organic compound levels such as acetone. Measurement of acetone from breath can allow a better diagnostic control of a patient’s diabetic condition than through the use of blood glucose measurements alone [30].

### Volatile Organic Compounds in Breath

Volatile organic compounds (VOCs) are either subtracted from inspired air (by degradation and/or excretion in the body) or added to alveolar breath as results of metabolic processes [31]. They exchange across the alveolar blood capillary membrane into exhaled breath [28]. There are many other sources of VOCs in human breath, including airway surfaces, inhaled room air, blood and peripheral tissues throughout the body. Some of these volatile organic compounds are the byproducts of biochemical reactions, whilst others might be produced for specific physiological roles such as cell-to-cell signalling. There are several classes of VOCs that can be measured in the exhaled breath. These include saturated hydrocarbons, unsaturated hydrocarbons, and oxygen-containing, sulfur-containing and nitrogen-containing compounds [25]. The saturated hydrocarbons include pentane aldehydes and ethane, which are formed by the lipid peroxidation of fatty acid components of cell membranes triggered by reactive oxygen species, while the unsaturated hydrocarbons include isoprene, and are produced by the melavalic pathway of acetoacetate from lipolysis or lipid peroxidation [32]. The oxygen-containing compounds, which include acetone are produced from decarboxylation of acetoacetate from lipolysis or lipid peroxidation [31]. The sulfur-containing compounds include ethyl mercaptane and dimethyl sulfide, and they are all produced from incomplete metabolism of methionine [33]. Last but not least, the nitrogen-containing compounds are produced during liver impairment and uremia [34]. So, analysis of VOCs in inspired air and alveolar breath is a useful research tool with crucial applications in clinical medicine [35]. As already mentioned in the text, different VOCs rather than acetone for diabetes are used as the biomarkers for different diseases in medicine. We are going to outline how other researchers have been able to diagnose diseases such as cancer, liver disease, kidney failure and so on using human breath. Phillips and coworkers’ research on VOCs showed that 22 VOCs are breath markers of lung cancer [36]. Methylated alkanes and selected alkanes have been shown to be able to distinguish lung cancer patients from healthy controls [37]. The rule of the lung cancer breath diagnosis is to check if a person’s breath contains more than 1 of the 11 VOCs with a concentration that is higher than the breath diagnostic cut-off. If the peak area of the 11 VOCs is >200, the patient is considered as a lung cancer patient [38].

Liver disease is one of the most prominent extra-oral causes of bad breath. It was found that dimethyl sulfide, acetone, 2-pentanone and 2-butanone were significantly higher in alveolar air of liver disease patients [39]. Impairment of the liver function increases the concentrations of these compounds, which have a characteristic smell of a rotten cabbage [40]. Other researchers used gas chromatography to demonstrate that the levels of all sulfur-containing molecules were elevated in the breath of patients with cirrhosis, even outside liver coma [41]. The characteristic sulfur odor could only be detected by the human nose, whereby physicians were sniffing the breath. The alveolar concentration of dimethyl sulfide is more than 30 nmol/mol or ppbv, which was observed in 25 of the 52 liver cirrhosis patients. Furthermore, hydrogen sulfide and methyl mercaptane have also been reported as possible mediators. However, in vitro experiments showed that the free -SH group of methyl mercaptane reacts with blood, which results in an irreversible binding and oxidation. In contrast, dimethyl sulfide is a neutral, stable molecule that can be transported from blood into alveolar air and be expired [39].

## 3. Acetone Metabolism

**Figure 1:**
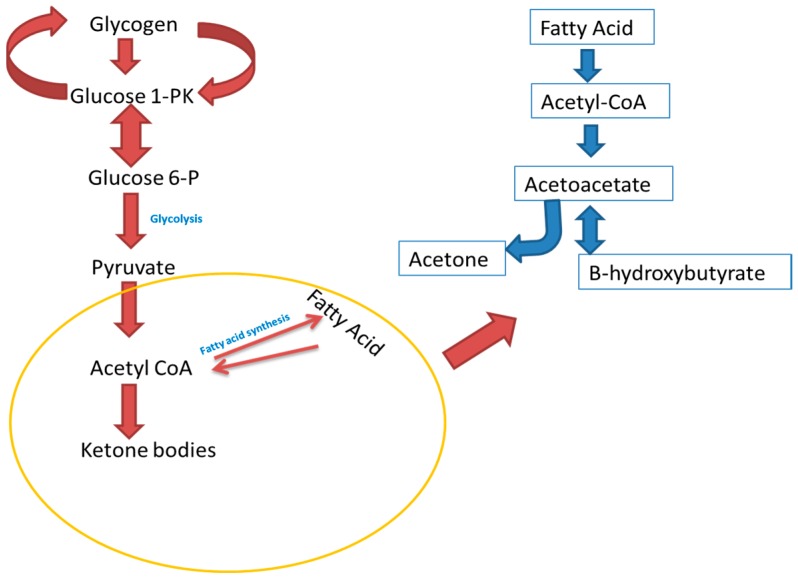
Ketone metabolism.

Acetone is an exhaled volatile organic compound that has been used as a biomarker for diabetes mellitus, especially in type 1 diabetes mellitus [19]. It is derived from oxidation of non-esterified fatty acids, which results in acetyl-CoA and ultimately acetoacetate through spontaneous decarboxylation or enzymatic conversion, as shown in Figure 1 [42]. The acetone that is produced travels through the blood and is excreted through urine, sweat and/or exhaled breath. For the exhaled breath, it has been found that the partition coefficient is 330 parts in the blood for every one part that leaves with expired air [20]. It has been found that quantification of acetone concentration in human breath, using breath analysis techniques, correlates strongly with acetone concentration in the blood and other ketone bodies such as beta-hydroxybutyrate. Furthermore, another study by Worrall et al. [35] has found that there is a correlation between blood glucose and volatile organic compounds. Thus, measurement of acetone from breath gives a better diagnostic control of a patient’s diabetic condition rather than through the use of blood glucose measurements alone.

Acetone levels in breath have also been used for detection and monitoring of abnormal concentrations of blood beta-hydroxybutyrate levels in diabetes mellitus [43]. It is reported that acetone is also emitted from the skin and its concentrations correlate with breath acetone and blood beta-hydroxybutyrate [44]. The odour of acetone in diabetic patients is used as a symptom of diabetic ketoacidosis, because acetone is produced as a direct byproduct of the spontaneous decomposition of acetoacetic acid. However, mild acidosis may be due to prolonged fasting or when the individual is on a ketogenic diet or a very-low-calorie diet [45]. Medical researchers have shown that the acetone concentration in exhaled breath from a normal individual is lower than 0.8 ppm, whilst that for a diabetic patient is higher than 1.8 ppm in seven diabetic and seven non-diabetic patients [46]. However, Anderson’s review has shown that different ranges of breath acetone can be found in different conditions. For example, adults on ketogenic diets can have elevated levels of up to 40 ppm. Children with epilepsy are treated with ketogenic diets to reduce the incidence of seizures, which leads to a high breath acetone concentration of 360 ppm. This is because fasting can cause the body to primarily utilize fats for energy production. These processes lead to 170 ppm of breath acetone. Poorly controlled diabetes can cause ketoacidosis which can increase breath acetone up to 1250 ppm [47].

## 4. Various Techniques Used in Diabetes Monitoring and Diagnosis

Monitoring of numerous volatile compounds in breath is a promising and expanding field. Techniques such as gas chromatography coupled to mass spectrometry (GC–MS), solid-phase microextraction (SPME), high-performance liquid chromatography (HPLC), selected ion flow tube mass spectrometry (SIFT–MS) and liquid chromatography-mass spectrometry (LC–MS) have provided highly selective analysis of VOCs in breath [48]. Although the GC and other mentioned analytical methods are highly sensitive and selective for diagnosis of diabetes mellitus, they are expensive and the issue of portability is particularly important considering that diabetes mellitus should be monitored and diagnosed in real-time for daily healthcare purposes [46].

GC–MS is a hyphenated technique which combines the separating power of gas chromatography with the detection power of mass spectrometry. It plays a fundamental role in determining how many components exist in a mixture, and their respective proportions. The mass spectrometric detector is commonly used to obtain the “fingerprint” spectrum of the molecule. Mass spectra provide information on the molecular weight, elemental composition, functional groups present, and in some cases, the geometry and spatial isomerism of the molecule [49].

With GC–MS, analysis of breath acetone requires sample preparation which includes acetone standard preparation and derivatization of acetone on the solid-phase microextraction (SPME) needle. The SPME derivatization is mostly used because the mass of acetone is too small and acetone is volatile [3]. Preparation of acetone standards takes about 20 min and having to derivatize it on the SPME takes 20 min [50]. The other disadvantage with sample preparation is the collection of breath from patients, which requires expensive Tedlarbags. The Tedlarbags can only store the human breath for not more than 6 h [3]. The GC–MS also suffers from temporal resolution. It takes considerable time, at least minutes if not tens of minutes, to separate fully the constituents of a gas mixture on a capillary column [51]. To analyse the acetone level in human breath using GC–MS, you need to get an extensive training from the manufacturer of the instrument or someone who has knowledge of GC–MS. Diabetes monitoring need a self-monitoring device that can be used in real-time for daily healthcare purposes.

Proton-transfer reaction mass spectrometry (PTR–MS) is a technique developed specifically for the detection of gaseous organic compounds in air [51]. In a PTR–MS system, primary ions, H_3_O^+^, react with neutrals under well-defined conditions. This is assured by allowing the H_3_O^+^ reactions to proceed within a flow-drift tube [52].Compared to GC–MS, PTR–MS delivers relative VOC concentrations with high sensitivity (down to parts per trillion) without sample preconcentration. Such preconcentration steps are time-consuming as already mentioned before. In addition, PTR–MS allows online measurements (over a full night, for example). The drawbacks of PTR–MS, on the other hand, include its inability to distinguish between substances having the same molecular weight. All substances identified by product ions with particular mass-to-charge ratios (*m/z*) must therefore be considered as possible contributors [53]. It requires a trained operator, or rather, a person who is scientifically literate. It does not suit illiterate people who are diabetic. Last but not least, PTR–MS is bulky; it is not suitable for a daily monitoring of diabetes mellitus [54].

Selected ion flow tube mass spectrometry (SIFT–MS) is also one of analytical techniques used for the real-time quantification of several trace gases simultaneously in air and breath. It relies on chemical ionization of the trace gas molecules in air/breath samples introduced into the helium carrier gas using H_3_O^+^, NO^+^ and O_2_^+^ precursor ions. Reactions between the precursor ions and trace gas molecules proceed for an accurately defined time, the precursor and product ions being detected and counted by a downstream mass spectrometer, thus effecting quantification. Absolute concentrations of trace gases in single-breath exhalation can be determined by SIFT–MS down to ppb levels with no sample collection and calibration required [55]. The shortfall of SIFT–MS as a potential instrument for monitoring diabetes is its inability to identify compounds in a mixture of gases. SIFT–MS, like GC–MS and PTR–MS, also requires a trained operator, and as already mentioned, diabetes affects old people who perhaps cannot read and write, so such instruments are not suitable for personalized monitoring devices. The other major and common drawback is portability.

Quantum cascade lasers (QCLs) are semiconductor lasers using transitions within the conduction band. Hence, the wavelength can be tailored by the thickness of the layer rather than being determined by the band-gap [56]. They can be fabricated to operate at any of a very wide range of wavelengths from ~3 μm to ~24 μm. They represent very promising sources for gas-sensing applications [57]. Systems based on widely tunable QCLs can be used to measure multiple gas species, and narrowly targeted systems can detect and measure gas concentrations in the parts-per-trillion range [58]. The principle of laser spectroscopy is based on the laser light being absorbed at the frequencies corresponding to the spectral absorption lines of the specific gases [59]. These absorption lines originate from the electronic, rotational or vibrational states of the molecules. The electronic-state transitions are much more energetic compared to the other two, and therefore can be detected using visible or near-UV wavelengths. The rotational and vibrational states can be probed using near- and mid-infrared light sources, due to much lower transition energies. The transitions occurring in the mid-infrared region, however, are much stronger, allowing much lower detection limits. QCL-based systems are finding application in the growing field of medical diagnostics. Such an application requires extremely fast sampling times, relatively small size and accurate results in order to avoid misdiagnosis; QCLs can fulfil these criteria [60]. However, there are some limitations to QCLs in diagnosis and monitoring of diabetes mellitus, which include selectivity required for practical use, and being currently limited by available technology to reach sufficient specificity.

Light-addressable potentiometric sensors (LPASs) are part of the field-effect-based biochemical sensors. They enable the monitoring of analyte concentrations in a spatially resolved manner on the sensor surface [61]. The LAPS principle was introduced in 1988 by Hafemann et al., and is based on the scanned light pulse technique (SLPT) (engestrom) with the intention to measure in aqueous solutions [62]. The great advantage of LAPS is its light-addressability, which makes the high-integrated level easily obtainable. LAPSs can obtain larger amounts of information than sensor arrays. Their large continuous surface can be used to determine the direction of gas as additional information for gas recognition. Image detection of respiratory gas based on LAPS is proposed as a development in the diagnosis of diabetes [63]. However, the drawbacks of LAPS in the diagnosis of diabetes mellitus as compared to chemoresistive sensors are as follows; it is bulky, thus not suitable for real-time point of care, and it requires a trained operator. Diabetes monitoring requires a self-manageable device.

For all the mentioned reasons, a portable diagnostic device with real-time monitoring as well as outstanding acetone sensitivity and selectivity should be developed. In this regard, semiconductive metal oxides (SMOs) are promising for diagnosis and monitoring diabetes mellitus non-invasively. This is due to their potential in real-time analysis, facile operating principle (resistivity change upon exposure of acetone to the SMO’s surface layers), simple device fabrication and ready miniaturization [64]. The basic operating principles as well as strengths and weaknesses of each method are summarised in Table 1.

As already mentioned, several online detection methods have been developed for the analysis of breath acetone samples using mass spectrometry, such as proton-transfer reaction mass spectrometry (PTR–MS), selected ion flow tube mass spectrometry (SIFT–MS) and gas chromatography–ion mobility spectrometry (GC–IMS) [54]. Among the developed analytical methods for VOCs, in particular, gas chromatography–mass spectrometry (GC–MS) is one of the most promising techniques. Due to the high sensitivity of typical VOCs, GC–MS has been widely employed in breath analysis. However, the concentration of VOCs in the typical breath sample is quite low, and thus a kind of sample preconcentration process, such as a cold-trap or adsorption trap method, is still necessary before analysis for the sensitive and accurate determination in most of the cases [3,24]. These methods provide high sensitivity; however, the techniques often require time-consuming processes. SPME is one of the most advanced sampling techniques for the GC analysis and has some advantages over conventional extraction methods, such as simple operation, being solventless and having easy automation [65,66]. Acetone breath concentrations taken from diabetic individuals using various analytical techniques are shown in Table 2.

## 5. Nanomaterial-Based Approaches for Detection of Acetone

The growing interest in nanotechnology has resulted in the identification of many unique physical and chemical properties associated with nanomaterials. The advances in nanotechnology are so profound that their impact echoes into society far beyond the boundaries of ordinary science [70]. The implementation of nanotechnology in the field of breath analysis with the chemoresistive sensors has been increased in recent years. The nanoscale size of these building blocks provides them with several qualities, such as large surface-to-volume ratios and unique chemical, optical and electrical properties. The large surface area of the nanomaterials provides highly active interfaces, thus increasing sensitivity and lowering the response and recovery times. Different nanomaterials have been exploited for VOC-sensing elements, including nanoparticles and nanowires of different materials. Furthermore, the dynamic range as well as the selectivity of the nanomaterial-based gas sensors can be tailored to accurately detect specific breath VOCs of a given disease [44,71].

Chemoresistive sensors based on semiconducting metal oxides have attracted attention for their potential for miniaturization and their low cost, simple fabrication, ease of use, good compatibility with microelectronic processes and reliability [72,73,74,75,76,77,78,79]. As already mentioned in Section 4, several methods such as GC–MS, IMS and SIFT–MS have been utilized to analyse trace compounds in the human breath. The major advantages of the mentioned techniques are high sensitivity, selectivity and low limit of detection. However, they are of high cost and not portable for diagnostic tools [78,79,80]. Chemoresistive sensors have been used mostly in the past years to detect sub-ppm acetone in human breath. It was found that a nanosized gamma iron sesquioxide (γ-Fe_2_O_3_) sensor is able to detect 1 ppm of acetone in the background of VOCs present in normal breath [81]. The screen-printed titanium dioxide (TiO_2_) sensor showed sensitivity for type 1 diabetes diagnosis [82]. Furthermore, pristine and Pt-functionalized tungsten trioxide (pt-WO_3_) hemitubes demonstrated a very high sensitivity and response to H_2_S and acetone gas [83]. WO_3_, particularly in its ε-phase, is promising for its selective and quantitative detection of acetone in the ppb concentration range. This is attributed to the spontaneous electric dipole moment of the ε-phase, which increases the interaction with analytes having high dipole moment (e.g., acetone) [20]. A portable diagnostic device with real-time monitoring, highly sensitive with a potential for miniaturization and pain-free implementation should be developed. Currently, chemoresistive SMOs have been receiving a great deal of attention for exhaled breath sensing in portable and real-time diabetes mellitus diagnosis. We have gathered recent publications in Table 3 on diabetes diagnosis by detection of acetone using SMO sensors. In addition, noble metallic catalysts such as Pd [84], Pt [82,85] and Au [86], as well as graphene-based catalytic materials and graphene oxide, have been integrated with nanostructured SMOs for selective acetone detection [87,88].

The rationale of using SMOs either as composites such as ZnO:Pt, ZnO:Nb, In/WO_3_-SnO_2_ or 2D C_3_N_4_-SnO_2_, doped such as WO_3_ decorated with Au and Pd, or pure metal oxides such as TiO_2_, 2D ZnO nanosheets or In_2_O_3_ nanoparticles, is because they offer a variety of advantages. These include low cost and facile fabrication [98,99]. Most reports on metal oxides for acetone sensing strengthen an emphasis on the facile synthesis of the SMOs [100,101,102,103]. Ai et al. used a microwave-assisted method, which is normally a cheap and fast method for material synthesis, to produce Fe_3_O_4_ nanoroses for gas-sensing applications [104]. The other interesting qualities of SMOs include their stability at a high working temperature, and last but not least, their abundance in nature.

The solid-state sensors are based on the adsorption/desorption or chemical reaction on the surface of the thin film material. This leads to change in the physical structure which is detected by the sensor device. The physical structure includes changes in temperature, refractive index, mass and more [20]. Considering the influencing factors on gas-sensing properties of metal oxides, it is necessary to reveal the sensing mechanism of the metal oxide gas sensor. The exact fundamental mechanisms that cause a gas sensor response are still controversial, but essentially, trapping of electrons at adsorbed molecules and band bending induced by these charged molecules are responsible for a change in conductivity [105]. The negative charge trapped in these oxygen species causes an upward band bending and thus a reduced conductivity compared to the flat band situation. When O_2_ molecules are adsorbed on the surface of metal oxides, they would extract electrons from the conduction band and trap the electrons at the surface in the form of ions. This will lead to a band bending and an electron-depleted region [76,106]. Reaction of these oxygen species with reducing gases or a competitive adsorption and replacement of the adsorbed oxygen by other molecules decreases and can reverse the band bending, resulting in an increased conductivity [107,108,109]. O^−^ is believed to be dominant at the operating temperature of 300–450 °C [110], which is the operating temperature for most metal oxide gas sensors.

Different factors influence the sensitivity of the metal oxides. These include the chemical composition, surface modification by noble metal particles, microstructure, humidity and temperature [76]. In addition to chemical composition, sensors based on the two mixed components tend to be more sensitive than the individual components. Kim et al. [59] in 2016 found a sensitive and selective acetone sensor using a WO_3_ nanofiber functionalized by Rh_2_O_3_ [96]. Furthermore, the conductivity response is also influenced by the efficiency of catalytic reactions with detected gas participation, taking place at the surface of the gas-sensing material. Therefore, control of catalytic activity of the gas sensor material is one of the most commonly used means to enhance the performance of gas sensors. However, in practice, the widely used gas-sensing metal oxide materials, such as TiO_2_, ZnO, SnO_2_, Cu_2_O, Ga_2_O_3_ and Fe_2_O_3_, are the least-active from a catalytic point of view [76]. The pure SnO_2_ thin film without any catalyst exhibits a very poor sensitivity (~3), confirming this statement. Moreover, as we continue with the factors that influence the sensor sensitivity, microstructure plays a crucial role. For example, a sensor’s sensitivity can be significantly increased by using materials with very small grain sizes, and this simulated result agrees well with the experimental observation. Lu et al. [111] have indicated that the SnO_2_-based sensor response to 500 ppm CO increases drastically if the particle diameter becomes smaller than 10 nm.

## 6. Limitations of Semiconducting Metal Oxides (SMOs)

As already mentioned above, generally the major limitations of SMOs include the following:

- sensitivity, a change of measured signal per analyte unit, that is, the slope of a calibration graph;
- selectivity, a characteristic that determines whether a sensor can respond selectively to a single analyte;
- stability, the ability of a sensor to provide reproducible results for a certain period of time. This includes retaining the sensitivity, selectivity, response and recovery time;
- durability, the ability to withstand damage due to temperature, chemical addition and so on;
- response time, the time required for the sensor to respond to a stepped concentration change from zero to a certain concentration value;
- recovery time, the time it takes for the sensor signal to return to its initial value after a stepped concentration change from a certain value to zero;
- room temperature operation and so on, the ability to detect gases at room temperature.

Nevertheless, there has been extensive research in trying to overcome the limitations of the SMOs in order to get good sensors. It was found that controlling the particle size and porosity of the material can enhance the sensitivity of the material. Numerous reports on SMOs reveal a high sensitivity when average grain size was reduced to several nanometers [112,113,114]. SnO_2_ and TiO_2_ mesoporous powders fabricated using self-assembly of a surfactant followed by treatment with phosphoric acid as well as conventional tin oxide powders with surfaces modified by mesoporous SnO_2_ show higher sensor performance than corresponding metal oxide powder materials, which have lower specific surface area [115,116]. Doping is also one way of improving the sensitivity of a SMO sensor that has been used for a long time. The sensitivity of metal oxide gas sensors can be greatly improved by dispersing a low concentration of additives, such as Au [117], Ag [118], Cu [119], Co, Pd [120,121], Pt [122] and F [52] on the oxide surface.

Two approaches have been developed to address the problem of selectivity. The first one is to prepare a material that is strictly sensitive to one analyte and has a low or zero cross-sensitivity to other analytes that are present in the working system. This is done by firstly optimising the working temperature, doping elements and their concentrations [115]. The second approach is based on the preparation of materials for discrimination between several analytes in a mixture. It is impossible to do this by using one sensor signal; therefore, it is usually done either by modulation of sensor temperature [123] or by using sensor arrays [124,125].

The issue of low stability can lead to problems of false alarms, uncertain results and the need to replace the sensor. Metal oxides with small grains, nanorods, nanotubes, nanowires and so on can be easily degraded due to their high reactivity. However, stability can be increased by calcination and annealing as the post-processing treatment and also reducing the working temperature of the sensor element. Synthesis of mixed oxides, and doping metal oxides with carbon nanotubes, have been reported to increase the stability of the sensor [115]. To overcome the response/recovery time of the sensor and room temperature operation, a patent has been filed that has a sensor with UV light to clean up the gas molecules so that the gas sensor is not saturated with breath [126].

The search for non-invasive diagnosis and monitoring of diabetes mellitus remains a worldwide goal. There are other complementary technologies being developed to help healthcare in monitoring diabetes mellitus non-invasively [127]. These include glucose-sensing technologies which are selective for glucose with a fast, predictable response to changing glucose concentrations [128]. This follows the non-invasive blood-glucose-sensing technique which measures in vivo blood glucose concentrations without collecting a blood sample. It uses near-infrared spectroscopy for accomplishing such a measurement. The concept is to pass a band of near-infrared radiation through a vascular region of the body and then to extract the corresponding blood glucose concentration from the resulting spectral information [129]. Different points of care testing (POCT) devices are available which are based on electrochemical or optical detection for measuring blood glucose [130].

## 7. Conclusions and Future Perspectives

The search for novel technologies for diagnosing and monitoring diabetes mellitus has been increasing tirelessly. Currently, there are sophisticated analytical methods for acetone detection, which include GC–MS, SIFT–MS, PTR–MS and so on, and they are being used in research and clinics. Clinical demonstrations showing correlation of blood glucose and blood ketones with breath acetone have been so far successful. Human breath analysis is a very promising method of disease detection due to non-invasiveness and simple diagnostic methods, especially for diabetes mellitus whereby a daily monitoring of blood glucose is required. Thus, monitoring breath acetone will be a cheap, non-invasive innovative technology for diabetes patients. The main aim is to achieve a fast, inexpensive and portable personalized device that could be implemented globally. The inclusion of nanoscale medical technologies into this framework will be a great success and allow better management and monitoring of diabetes mellitus. These advances will empower patients and the general population by providing them with personalized devices for monitoring, thus drastically reducing healthcare costs and potentially leading to significantly improved healthcare.

## Figures and Tables

**Table 1:** Comparison of breath acetone analysis techniques.

Technique	Principle	Detection Limit	Advantages	Disadvantages
GC–MS	Separate and analyse compounds by MS using chromatographic column (polar or non-polar)	Ppb and ppt levels	Highly selective and sensitive	Preconcentration steps, bulky, long sampling time, need for standards and requires trained operator
PTR–MS	Analysis of ionized molecules of target analytes by reaction with H_3_O^+^ MS	Low ppb levels	Real-time analysis	Lack of specificity, Narrow range of detectable compounds, bulky and requires trained operator
SIFT–MS	Analysis of ions produced by the reaction analytes and precursor ions (H_3_O^+^, NO^+^ or O_2_^+^) by quadrupole MS	Low ppb and ppt levels	Real-time, capability of ppt detection, broad range of detection	Cannot identify compounds, bulky and requires trained operator
QCL	Electrons are recycled from period to period, containing each time to the gain and the photon emission	Low ppb levels	Real-time analysis, potential for portability and miniaturization	Selectivity required for practical use and currently limited by available technology to reach sufficient specificity
LPAS	Analysis of trace gases. It uses the photoacoustic effect, the conversion of light to sound in all materials (solid, liquids and gases)	Ppt–ppb levels	Real-time analysis	Bulky, requires trained operator
SMOS-based chemoresistive sensors	Measures resistivity changes based on thinning or thickening the depletion layer of n-type SMOSs and hole accumulation layer of p-type SMOSs around the surface when exposed to oxidizing or reducing ambient gas	Ppm, ppb and ppt levels	Real-time analysis, portable, inexpensive and miniaturization	Relatively low sensitivity and less selectivity

**Table 2:** Techniques and theirdetection ofbreath acetone concentration.

Technique	Acetone Concentration	Reference
GC–MS	0.049 ppb	[67]
	0.22–3.73 ppb	[3]
	06.95–145.99 ppb	[24]
	0.195–0.659 ppm	[54]
PTR–MS	0.19–1.3 ppm	[67]
	50 ppb	[68]
	200–2000 ppb	[69]
SIFT–MS	1–20 ppm	[8]
	293–870 ppb	[69]

**Table 3:** Recent publications on chemoresistive SMO-based exhaled breath sensors for potential use in diagnosis of diabetes mellitus using acetone as a biomarker.

Material	Sensitivity (Response) (ppm)	Detection Limit (ppm)	Response/Recovery Time (s)	Operating Temperature (°C)	Reference
ZnO:Pt	188	1000	45	400	[89]
ZnO:Nb	224.0	1000	56	400	[89]
PrFeO_3_	234.4	500	6.1	180	[90]
CdNb_2_O_6_	2	10	9	600	[91]
In/WO_3_-SnO_2_	66.5	50	2.12	200	[92]
2D C_3_N_4_-SnO_2_	11	67	7	380	[93]
TiO_2_	15.24	500	9.19	270	[94]
2D ZnOnanosheets	106.1	500	-	300	[95]
WO_3_ decorated with Au and Pd	-	1000	6	300	[96]
In_2_O_3_ nanoparticle	21.5	1000	2	250	[97]
